# Supplementary material for: Identification of photoperiod-induced specific miRNAs in the adrenal glands of Sunite sheep (Ovis aries)
Source: Front Vet Sci. 2022 Jul 22;9:888207. doi: 10.3389/fvets.2022.888207 (PMC9354845; doi:10.3389/fvets.2022.888207)
Supplement: Supplementary file 1 [file Data_Sheet_1.ZIP › Supplementary file/miRDeep2 file.zip!/miRDeep2 file/Know_Structure/SP42A3/oar-mir-1197.pdf]

miRBase precursor : oar-mir-1197
Total read count : 42
oar-miR-1197-5p read count : 0
oar-miR-1197-3p read count : 37
remaining reads : 5

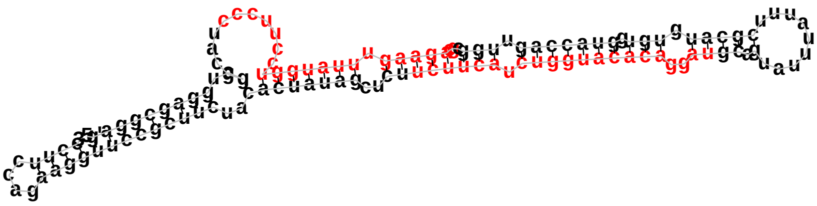

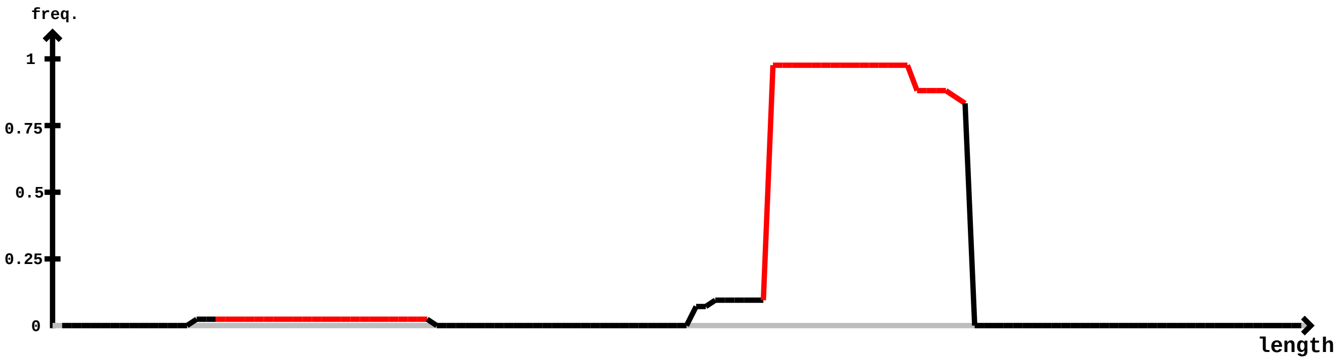


```
                                                                          oar-miR-1197-3p
                oar-miR-1197-5p
5'- gaggcgaggucggcaucccuuccugguauuugaagacgcgguugaccaugguguguacgcuuuauuuaugacguaggacacauggucuacuucuucucgauaucacaucuucgccuuggaagaccuucc -3'   exp
    (((((((((..(...........(((((((.(((((...(((.(((((((.(((.(((((.........).))))..)))))))))).))))))))..))))))))..)))))))))((((....))))   reads  mm
    .............caucccuuccugguauuugaagacg...........................................................................................   1      0
    .................................................................uuaugacguaggacacauggucu.........................................   3      0
    ...................................................................augacguaggacacauggucu.........................................   1      0
    .........................................................................uaggacacauggucuacuu.....................................   1      0
    .........................................................................uaggacacauggucuacuuc....................................   1      0
    .........................................................................uaggacacauggucuacuucu...................................   35     0
```
